# A digital image colorimetry system based on smart devices for immediate and simultaneous determination of enzyme-linked immunosorbent assays

**DOI:** 10.1038/s41598-024-52931-6

**Published:** 2024-01-31

**Authors:** Shaghayegh Mirhosseini, Aryanaz Faghih Nasiri, Fatemeh Khatami, Akram Mirzaei, Seyed Mohammad Kazem Aghamir, Mohammadreza Kolahdouz

**Affiliations:** 1 School of Electrical and Computer Engineering, College of Engineering, University of Tehran, Tehran, Iran; 2 Urology Research Center, Tehran University of Medical Sciences, Tehran, Iran

**Keywords:** Bladder cancer, Prostate cancer, Laboratory techniques and procedures

## Abstract

Standard enzyme-linked immunosorbent assays based on microplates are frequently utilized for various molecular sensing, disease screening, and nanomedicine applications. Comparing this multi-well plate batched analysis to non-batched or non-standard testing, the diagnosis expenses per patient are drastically reduced. However, the requirement for rather big and pricey readout instruments prevents their application in environments with limited resources, especially in the field. In this work, a handheld cellphone-based colorimetric microplate reader for quick, credible, and novel analysis of digital images of human cancer cell lines at a reasonable price was developed. Using our in-house-developed app, images of the plates are captured and sent to our servers, where they are processed using a machine learning algorithm to produce diagnostic results. Using FDA-approved human epididymis protein of ovary IgG (HE4), prostate cancer cell line (PC3), and bladder cancer cell line (5637) ELISA tests, we successfully examined this mobile platform. The accuracies for the HE4, PC3, and 5637 tests were 93%, 97.5%, and 97.2%, respectively. By contrasting the findings with the measurements made using optical absorption EPOCH microplate readers and optical absorption Tecan microplate readers, this approach was found to be accurate and effective. As a result, digital image colorimetry on smart devices offered a practical, user-friendly, affordable, precise, and effective method for quickly identifying human cancer cell lines. Thus, healthcare providers might use this portable device to carry out high-throughput illness screening, epidemiological investigations or monitor vaccination campaigns.

## Introduction

The first rapid detection method, point of care (POC), was based on lateral flow assays (LFA) for pregnancy tests back in the 1970s^1,2^. LFAs rely on the color change caused by the reaction between the target biomolecule and its biomarker using labels such as gold nanoparticles, fluorescence particles, etc.^3^. The color change created in LFAs is visible to the naked eye. However, LFAs have disadvantages, including low accuracy, the need for large volumes of biomolecules and biomarkers to receive the faintest eye-detectable signal, qualitative responses instead of quantitative or quasi-quantitative outputs, and the inaccuracy of naked-eye detection, or in other words, the fallacy of interpretative subjectivity^4–6^.

The aforementioned method, limited to single-use, is expensive and has a tedious procedure^6^. To resolve the challenges associated with the LFAs, an enzyme-linked immunosorbent assay (ELISA) was proposed. This method of diagnosis can detect multiple samples simultaneously via all 96 plates, making laboratory diagnosis swifter and less expensive. In addition, it uses a color-changing mechanism based on enzymatic reactions that reduces the volume of biomarkers required for the minimum detectable signal^7^.

The application of the optical absorption mechanism in ELISA readers dramatically increases the detection accuracy compared to the LFA^1,6,8,9^. Despite the numerous diagnostic advantages of ELISA, the ELISA reader’s reliance on large light absorption equipment as well as its non-portability present drawbacks which limits availability of ELISA-based diagnostics in well-equipped modern laboratories. As the gold standard in various tests, the FDA-approved 96-well plates’ structure cannot be altered in order to enhance their performance. Replacing the optical absorption system with alternative mechanisms, such as a digital ELISA system with smaller device’s dimensions and highly accurate detection at the femtomolar scale, or amplification of the color change signal through the surface plasmon enhancement effect, offers a solution to reduce the size of the ELISA while improving accuracy^10,11^. Recently, several POC ELISA methods have been created based on microfluidic platforms or paper-based devices to increase accessibility in resource-constrained or rural places^12–18^. One of the most advanced innovations involves the integration of a microfluidic ELISA platform with a smartphone dongle capable of performing multiple functions, including pumping and imaging of a sandwich ELISA’s silver precipitation readout^19^. Widely used in clinical labs, various multi-well plate ELISA test implementations have already received FDA approval, which reduces the cost of future regulatory requirements for modifications. The conventional ELISA reading methods use expensive and bulky scanning-based spectrophotometry and requires a stable power supply to scan each individual well, severely restricting its use in resource-constrained or remote locations. Additionally, image processing techniques, utilizing digital cameras or scanners, provide an alternative method to measure color and read the concentration of all 96 ELISA plate wells, analyzing them in one image. Alternatively, other imaging-based detection techniques involve scanners^20^ or charge-coupled device (CCD) cameras^21–23^ to take one picture of the 96-wells. The challenge of miniaturization of these rapid optical imaging methods into a self-contained, reliable hand-held unit with minimal optical aberrations arises from the necessitation of large fields of view (FOV) images covering the full extent of the plate’s surface (127 $\times$ 85 mm^2^) simultaneously. The ideal POC well plate reader platform would feature a smart user-friendly interface, real-time on-site image processing, wireless connectivity, instant reporting and sharing, spatiotemporal labeling and archiving, and the incorporation of visualization of diagnostic outcomes for applications such as telemedicine and POC screening. While the aforementioned imaging devices increase the dimensions of the devices which fail to relate to their portability, smartphones equipped with cameras and processors have revolutionized image processing capabilities and spawned a new generation of diagnostic systems, offer portability, affordability, real-time image processing, processing and interpretation of the images, and increased accessibility and availability of diagnostic amenities. With the integration of cameras into smartphones, professionals in the healthcare industry can conveniently take images for diagnostic goals, eliminating the requirement of specialized equipment, thereby reducing the overall expenses. This portability also enables telemedicine applications by allowing professionals to remotely diagnose patients based on the captured images, providing immediate feedback, and providing swift choices.

Recent developments in wireless communication technology and consumer electronics have sparked a revolution in biomedical imaging, sensing, and diagnostics^3,24,25^. Mobile cellphone-based devices have evolved into microscopy and sensing platforms for various applications^3,24–34^, including blood analysis^35–38^, bacterium identification^39–41^, single-virus imaging^42,43^, DNA imaging and sizing^44,45^, chemical sensing^46–49^, and biomarker detection^18,33,34,50,51^. These methods use the mobile internet platform to transfer and share data, analyze results, etc. These features provide an attractive context for introducing ELISA readers which are based on image processing on the mobile platform^52–56^.

Herein, we demonstrate a cost-effective and portable ELISA reader for microtiter plate (MTP) reading. This ELISA reader is based on colorimetry using images captured by a smartphone and then processed on a server. At the same time, similar tests were performed using an FDA-certified calibrated ELISA reader, Optical absorption EPOCH microplate readers and Optical absorption TECAN microplate readers for comparison.

## Results and discussions

In order to develop a portable and miniaturized version of an Enzyme-linked Immunosorbent Assay (ELISA) for applications in telemedicine, underdeveloped areas, and rapid clinical test evaluation, we constructed a 3D printed device. By utilizing a smartphone and the custom-built opto-mechanical platform mentioned earlier, we captured images of a typical 96-well plate while testing PC3, 5637, and HE4 cell-lines. These findings demonstrated that our portable and cost-effective device can deliver performance on par with a traditional FDA-approved ELISA reader, providing users with precise diagnostic outcomes within minutes. Our system exhibited comparable findings for various illnesses typically assessed using conventional ELISA methods. Our study utilizes a learning-based model to correlate cancer cell lines' concentration with R, G, and B parameters. The Lab-on-a-phone device we developed offers advantages such as high sensitivity, accuracy, user-friendly operation, and ubiquitous detection capabilities. We maintain uniform irradiance conditions in colorimetry through the use of a TFT LCD. Employing adaptive boosting-based machine learning algorithms enhances our methodology by focusing on significant features, ensuring accurate and reliable results.

**Figure 1:**
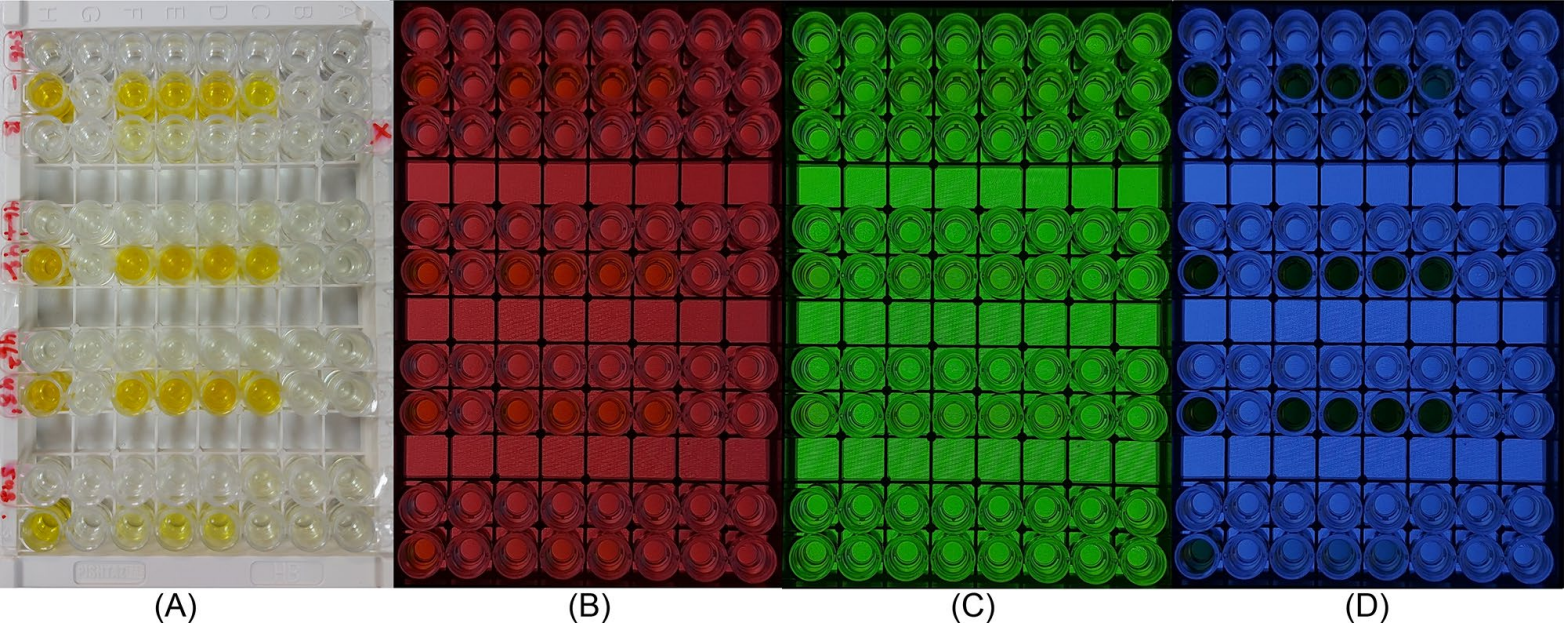
Images of a 96-well plate container using a smartphone camera (**A**) optical image under normal light, optical image while the LCD was adjusted to (**B**) red light Red, (**C**) Green light, and (**D**) Blue light.

**Figure 2:**
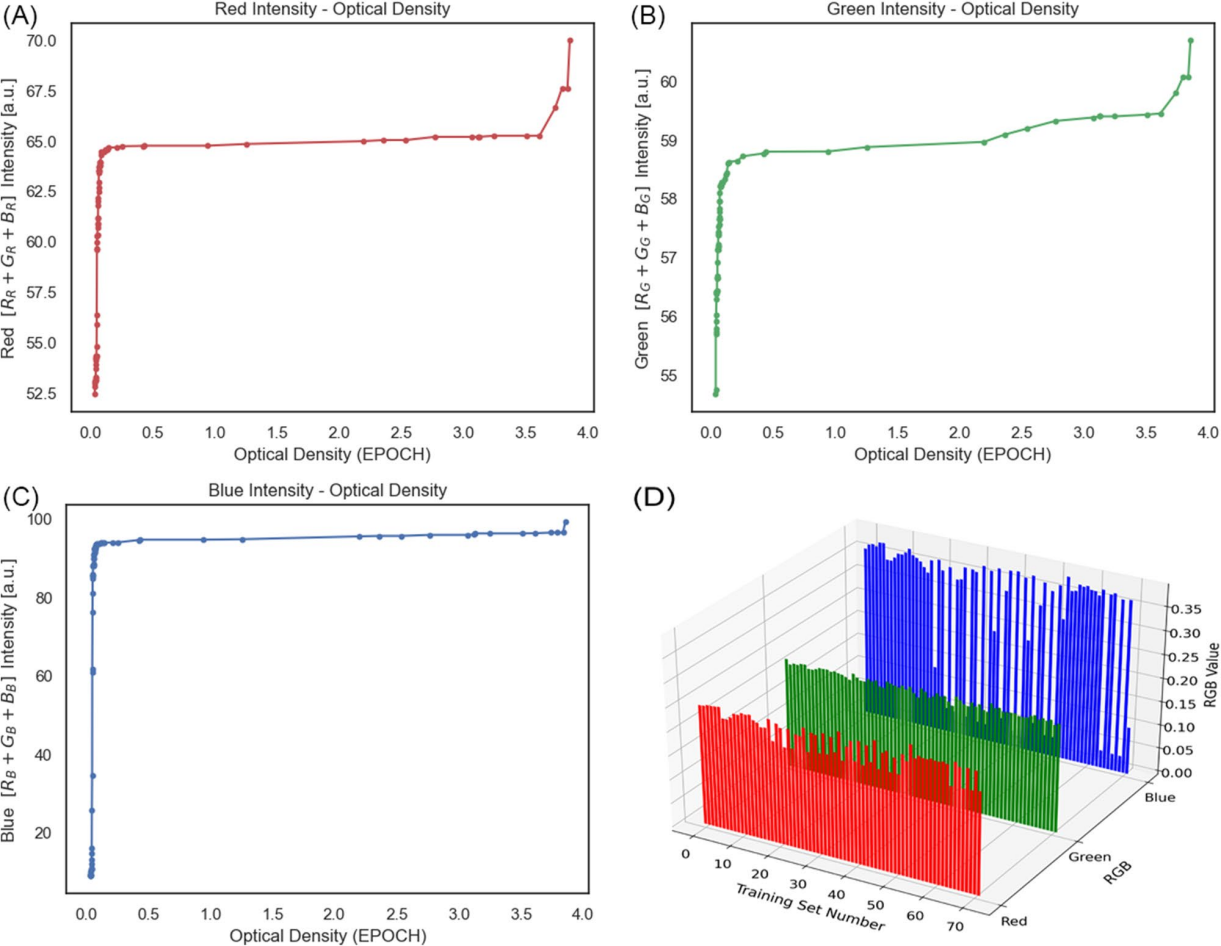
RGB Intensities Compared to Epoch Optical Density (**A**) R (r, g, b), (**B**) G (r, g, b), (**C**) B (r, g, b) and (**D**) All Inputs.

The workflow begins by filling the 96-well plate with the test samples mentioned previously. Subsequently, the ELISA plate is inserted into the device, and images are captured in three wavelengths: green, blue, and red, as depicted in Fig. 1. Next, three RGB values are obtained for each well, representing the average pixel intensity within the cropped circle of each well. To ensure accurate evaluation of the RGB values, it is crucial to maintain consistent test conditions, and carefully assess and consider the effects of light interferences and differences in light paths. Figure 2 depicts the RGB input values and their corresponding Epoch Optical Densities.

Table 1 compares the results taken from our mobile phone-based ELISA reader with two commercially available ELISA readers. One of these commercially available readers is the EPOCH, which measures 32 $\times$ 32.3 $\times$ 39.54 cm^3^ with a weight of 11.34 kg. In comparison, our device’s design reduces the overall volume of the reader instrument (141 × 222 × 250 mm^3^) and the weight considerably.

**Table 1:** Comparison between the Optical Density (OD) of the Smartphone-Based Colorimetric Device and the Optical absorption microplate readers for Cancer Cell lines Concentration Detection.

Sample	Elisa reader method		Smartphone-based method
	EPOCH microplate readers	TECAN microplate readers	Digital image colorimetry device
He4	0.074	0.068	0.073
	0.058	0.049	0.058
	0.059	0.054	0.064
	0.054	0.055	0.053
	0.122	0.116	0.116
	3.738	2.665	3.546
PC3	0.660	0.718	0.681
	0.672	0.691	0.672
	0.522	0.548	0.500
	0.818	0.832	0.824
	0.324	0.310	0.323
5637	0.672	0.931	0.674
	0.363	0.391	0.362
	0.383	0.404	0.377
	0.328	0.369	0.327
	0.895	1.196	0.895
ACHN	0.171	0.167	0.173
	0.175	0.184	0.175
	0.176	0.194	0.175
	0.255	0.270	0.255
	0.406	0.342	0.403
	1.127	1.133	1.118

Regression is an effective and supervised learning technique for modeling and creates a rational, mathematical relation between inputs called features, their impacts, and an output to be able to later make approximate and accurate predictions based on the same relation. Accordingly, we extracted RGB values from each image and the outputs are their corresponding optical densities. 72 cancer cell lines were used as sample images. Consequently, their corresponding extracted RGB values create the training set matrix; in other words, each RGB value is a training example, and the whole stack is the training set. R, G, and B are each a feature in the training set. X_i, j_ refers to the i_th_ training example and the j_th_ feature. The values of the training example and features are 1 ≤ i ≤ 71 and 1 ≤ j ≤ 3, which both are -1 shifted since in Python indices start from 0. Also, according to the hypothesis formula, there needs to be a bias, so a column of 1 s needs to be added.

The input matrix is as below:

1 $$X= \left[\begin{array}{cccc}{X}_{0}& R& G& B\\ 1& {r}_{0}& {g}_{0}& {b}_{0}\\ \vdots & \vdots & \vdots & \vdots \\ 1& {r}_{71}& {g}_{71}& {b}_{71}\end{array}\right].$$

The output matrix is as follows:

2 $$Y= \left[\begin{array}{c}Optical \,Densities\\ {y}_{0}\\ \vdots \\ {y}_{71}\end{array}\right].$$

The hypothesis or the predicted output array is:

3 $$h= {\theta }_{0}{X}_{0}+ {\theta }_{1}R+ {\theta }_{2}G+ {\theta }_{3}B.$$

Accordingly, the coefficient matrix is:

4 $$\Theta =\left[{\theta }_{0}, {\theta }_{1},{\theta }_{2},{\theta }_{3}\right].$$

So, in order to acquire the coefficient matrix, the algorithm is as follows:

5 $${\Theta }^{T}X=Y$$

6 $$\Theta ={\left({X}^{T}X\right)}^{-1}{X}^{T}Y.$$

Moreover, for estimating the coefficients in *Θ*, the Root Mean Square Error (RMSE) loss function needs to be optimized. By minimizing the RMSE, the coefficients are estimated.

7 $${\text{RMSE}}= \sqrt{\frac{1}{{\text{N}}}\sum_{{\text{i}}=1}^{{\text{N}}}{\left(\left|{\text{y}}-{\text{h}}\right|\right)}^{2}}.$$

An RMSE solver was used to minimize the loss function. For the regression algorithm, a machine learning code was written using Python libraries. Primarily, the *Θ* matrix was gathered by using the known values of optical densities, the “Epoch Optical Densities” and when the matrix was created, other data were then predictable.

To check the accuracy, two different correlation techniques were implemented. Firstly, Pearson’s Correlation (r) followed by Spearman’s Correlation (ρ). The first one indicates the linear correlation between the predicted result and the Epoch optical density, while the other measures the trends or, as it is called, ranks. The results were 0.993 and 0.834, respectively. When Pearson’s Correlation is near 1 (or − 1), it indicates a total linear correlation, and when it is more than 0.5, it means that there is a robust linear correlation and accordingly 0.993 is almost a solid linear correlation. 0.834 for Spearman’s correlation means that 0.834 of the trends are followed, meaning that either increasing or decreasing one results in the same transformation in the other. The more the input, the more the optical density and vice versa.

8 $$Lo{g}_{10}OD= {\theta }_{1}R+ {\theta }_{2}G+ {\theta }_{3}B,$$

where θ_1_, θ_2_, and θ_3_ are the coefficients of Red, Green, and Blue, respectively which, as explained, were the unknown quantities of the hypothesis equation. By carrying out the regression algorithm as stated above, the coefficients were figured out. However, for a more accurate result, more data were created from the given data before the regression was implemented.

**Figure 3:**
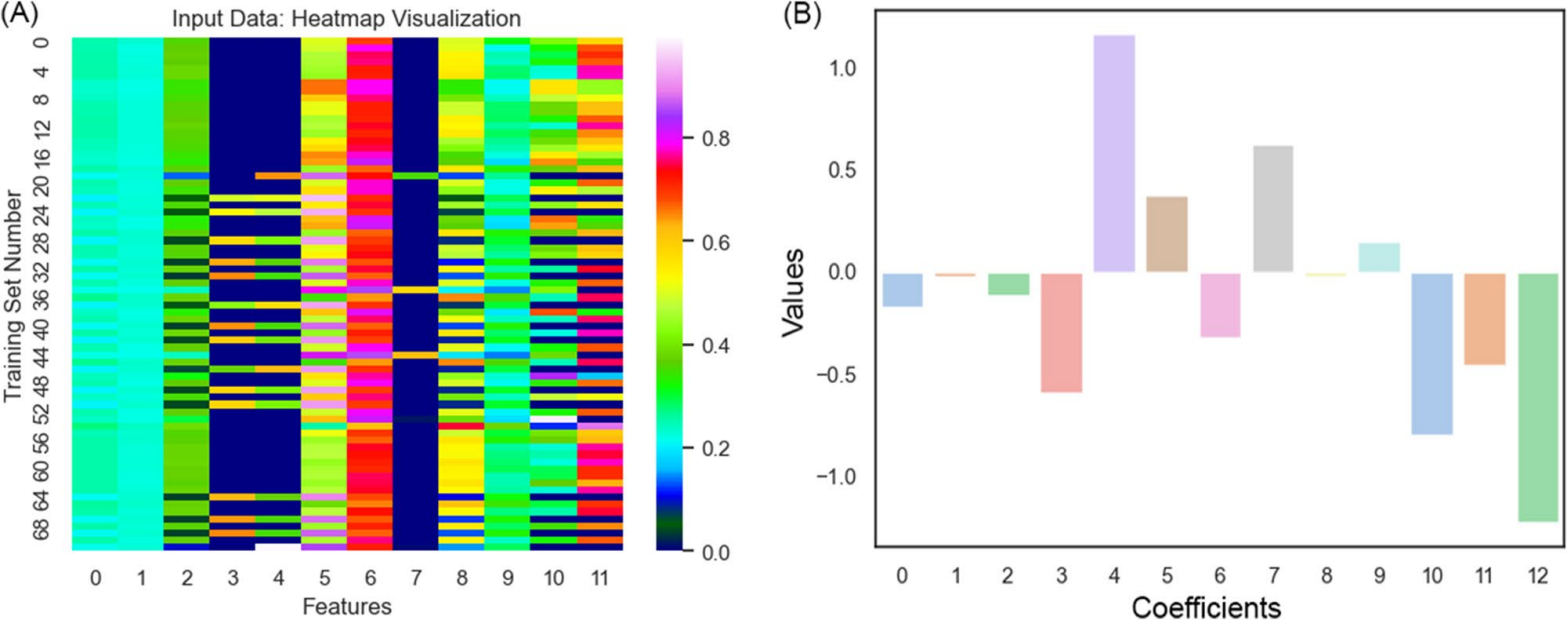
(**A**) Extracted features of images, (**B**) Representation of the regression coefficients.

The feature engineering was carried out and for each new feature, the engineering was implemented by a newly given formula, and then the columns with ‘all-zero’ elements were deleted, and the number of features was boosted from the initial 3 (R, G, and B) to 12. Figure 3A is the demonstration of the same matrix, with 12 features and 72 data examples. Looking at the picture, row-wise, the heatmap visualization depicts that row 0 has an R and G near 0.2 and a B near 0.3, and the rest in a similar manner.

Furthermore, a column of 1 s was added to the left side of the matrix (Column 0) for the bias, and post-regression, the now-known coefficients were as the bars displayed in Fig. 3 Image (B).

### Cross-validation

**Figure 4:**
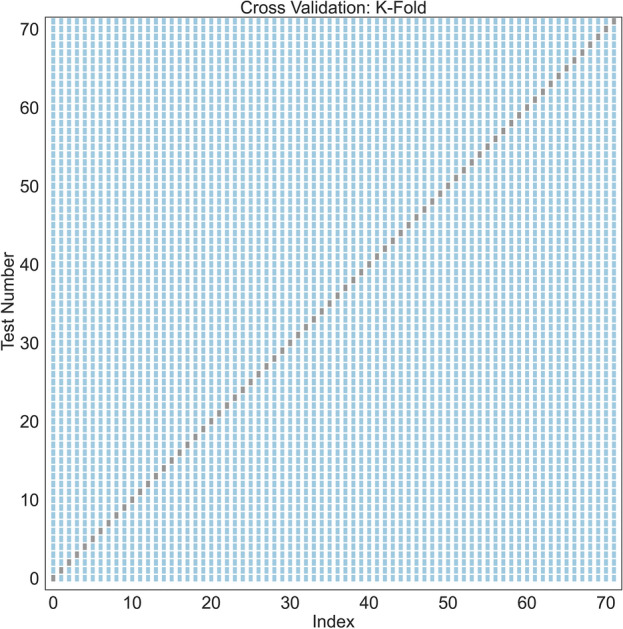
K-Fold validation used for cross-validation.

K-fold cross-validation is an effective method for assessing a model's predictive capacity. Utilizing this technique, datasets are divided into K subsets, allowing the model to undergo training K times, each with a distinct training set. In the context of the Cross-Validation section, a k-fold validation approach was employed, iterating through the dataset 72 times (k = 72), resulting in 72 testing instances and 5184 training instances. During each iteration, one example set was strategically excluded from the entire matrix. This systematic approach involves designating one fold as the testing set, while the remaining k-1 folds function as the training model, a process repeated k times to ensure each fold acts as the testing set once. This careful cycling through various subsets for the training and testing sets provides a thorough and accurate assessment of the model's performance. The test data set was as Fig. 4*.*

**Figure 5:**
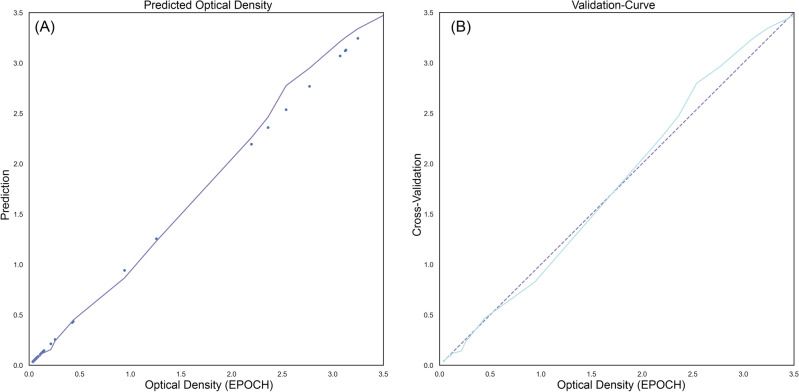
(**A**) Predicted Optical Density and (**B**) Validation Curve.

The RMSE, an essential metric measuring the disparity between actual experimental data and the predicted values (as denoted by Eq. (7)) to determine the accuracy of the predictive abilities of the model, was computed for each training and testing set, yielding values of 0.0817 and 0.0877, respectively. A model with smaller RMSE fits the data better. Figure 5 displays a graphical representation of the predicted values from the training and testing sets compared to the actual experimental data, revealing the presence of a good harmony between them. Our model shows precise prediction as evidenced by the low RMSE values for train and test predictions. In other words, the predicted values were relevant to the actual experimental data, showcasing the model’s accuracy and reliability. Figure 5 both demonstrates and confirms the relatively low RMSE and proves that since the training and testing errors are approximately close as well as not being exactly on the experimental data, there is no overfitting or high variance involved. In other words, the model is a balanced model when comparing the training and testing errors since the proximity of these errors which are roughly similar but not exactly equal to the experimental values which is evidence of the absence of high variance or overfitting. The results of the k-fold cross-validation coupled with the RMSE are satisfactory, valid, and have the ability to become generalized so that the model can extend beyond the training set and effectively handle the un-trained data.

To conclude Table 2 represents the general characteristics of the optical device:

**Table 2:** Characteristics of the digital image colorimetry system.

Feature	Description
Application	Molecular sensing, disease screening, nanomedicine applications
Analysis type	Colorimetric microplate reader for digital image analysis
Diagnostic tests	HE4, PC3, and 5637 ELISA tests
Accuracy of tests	HE4: 93%, PC3: 97.5%, 5637: 97.2%
Comparison tests	Optical absorption EPOCH microplate readers, Optical absorption Tecan microplate readers
System components	Handheld cellphone (Samsung A71), 7-inch LCD (1024 × 600, HDMI, IPS Panel), Raspbian operating system, 3D printed black box (141 × 222 × 250 mm^3^), Microplate holder (MDF with 2.8 mm black bases), Machine learning algorithm
Device size	7-inch LCD; Black box dimensions: 141 × 222 × 250 mm^3^
Image capture method	Smartphone camera capturing three images with red, blue, and green lights
Image processing	Linear regression model for RGB code extraction and machine learning stages
Advantages	Practical, user-friendly, affordable, precise, effective, portable, simultaneous detection, label-free, low-cost
Environmental considerations	Eco-friendly due to reusability and absence of third-party utilization
Drawbacks	Camera quality dependency, potential connectivity issues, smartphone battery dependency, susceptibility to external influences, inconsistency among different smartphone models

In comparison to previous studies that utilized paper strips or achieved 89.5% and 84.2% accuracy, the same device exhibited superior accuracy and eco-friendliness due to the absence of third-party utilization and its reusability^15,57^. Furthermore, its direct utilization of samples, without the need for labeling with nanoparticles or other materials, provided a competitive advantage over similar systems with comparable objectives^15^. The absence of third-party involvement allowed each 96-well plate to accommodate different biomarkers or cancer cell lines, enabling simultaneous detection of every well. Also being cost-effective is a major advantage.

In a related study by Berg et al., Light Emitting Diodes (LEDs) were employed as the light source. However, in this paper, a Liquid Crystal Display (LCD) was used, offering the advantage of controlled and homogeneous illumination, thus enhancing the system's performance^9^.

The simultaneous detection of multiple samples, a notable advantage, facilitates efficiency and time savings in diagnostics. This approach is label-free, eliminating the need for additional markers or labels. The low-cost nature of the method makes it accessible for widespread use. Another advantage is portability, which enables on-site testing in various environments.

There are, however, drawbacks that come with it. The quality of the camera used becomes a crucial factor, influencing the accuracy of results. There could be problems with connectivity that affect data transfer. Dependency on smartphone batteries poses a potential drawback, requiring a stable power source. These systems could be susceptible to outside influences because of their delicate design. Additionally, there might be inconsistency among different smartphone models, affecting the reliability, standardization, and accuracy of the results. Also, a drawback of colorimetry lies in its potential lack of sensitivity when detecting substances present in low concentrations.

The use of Digital Image Colorimetry Systems introduces some challenges. These encompass the necessity for precise color measurement and calibration across diverse devices and environments and the complexity of adapting color measurements to fluctuating environmental conditions such as lighting and temperature. This poses difficulties in maintaining consistent and reliable colorimetry. Efforts to address these challenges have seen notable advancements, mainly through integrating AI-driven algorithms, leveraging machine learning to compensate for environmental variations, and thereby enhancing the robustness of colorimetric measurements.

## Material and methods

### Smartphone-based colorimetric device

**Figure 6:**
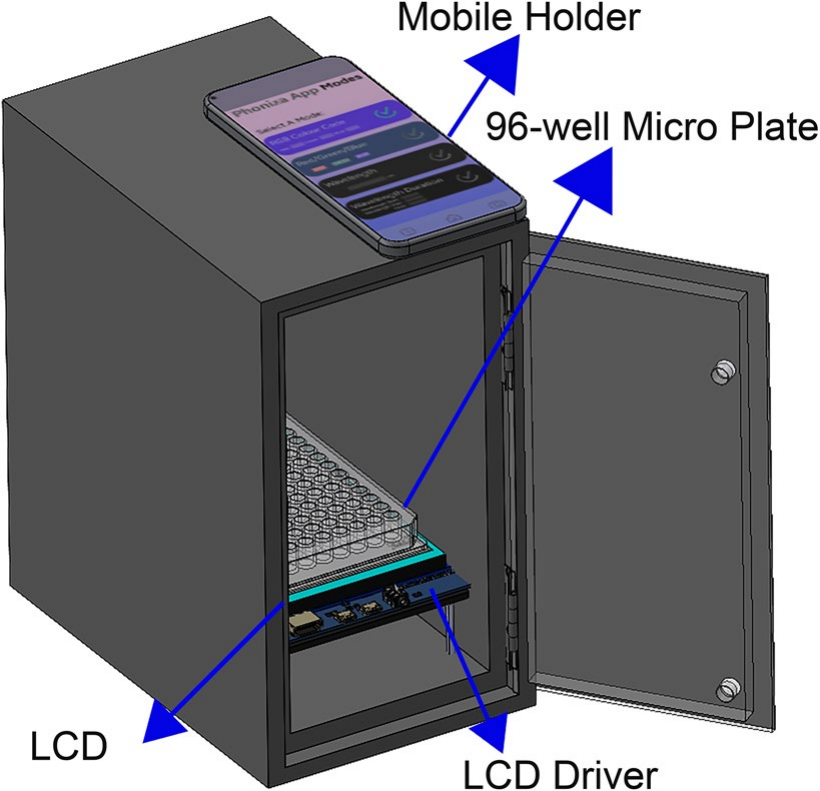
Schematic view and optical density detection for different Cancer cells of the cell phone-based ELISA colorimetric reader.

A schematic of the smartphone-based colorimetric device is shown in Fig. 6. To protect the system from outside light, a black box with dimensions of 141 × 222 × 250 mm^3^ was made by 3D printing. Digital images are taken through the mobile cellphone (Samsung A71) via a hole contrived at the top of the box. Solutions containing the cancer cell lines are placed in a 96-well microplate on a microplate holder made of MDF with 2.8 mm black bases placed on top of the LCD (see Fig. 6). A 7-inch LCD with specifications; 1024 × 600, HDMI, IPS Panel was connected to electrical circuits and placed under the microplate holder. The reason for choosing this type of display is its accuracy and large angle of view. The Raspbian operating system was installed on the mainboard and connected to the monitor via the mentioned HDMI cable. The filter renders the LCD light, which then passes through the 96-well ELISA plate, and reaches the smartphone camera lens. The LCD light is set in the visible light wavelength range, and for each ELISA plate, three images with red, blue, and green lights are captured by the smartphone camera and transmitted to a computing server (Fig. 1). The processing program extracts a three-digit (RGB) code for each well from each green, blue, and red image. Then, the linear regression model is used for the image processing and machine learning stages.

### Cell lines and cell culture sample preparation

**Figure 7:**
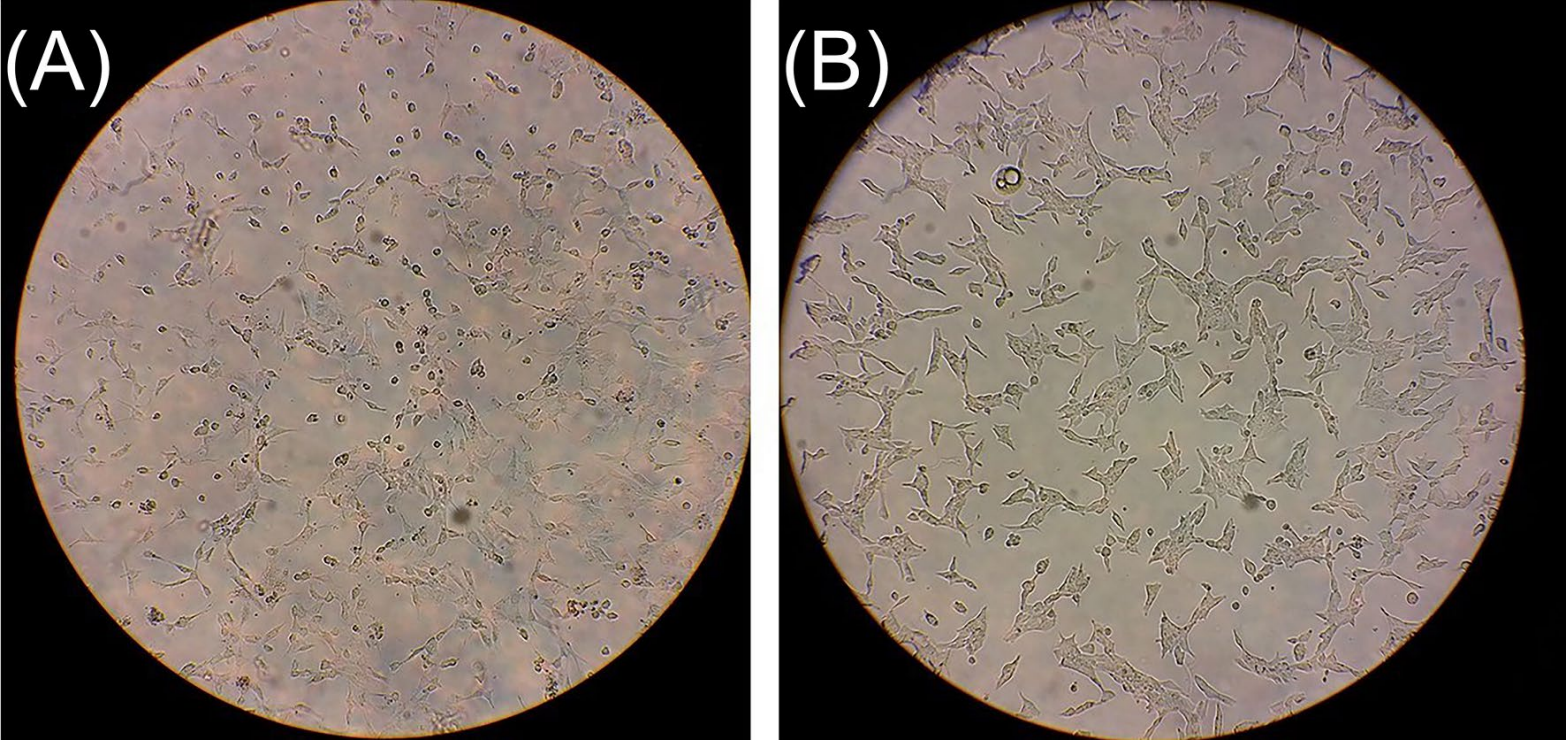
Photographs of human prostate cancer cell line, LNCaP (**B**) and PC3 (**A**) cells were photographed at magnification, × 400.

Human cancer cell lines^58^, HE4 (Human Epididymis Protein of Ovarian Cancer), PC3 (Bone Metastasis of Grade IV of Prostate Cancer), LNCap (an epithelial cell line derived from a human prostate carcinoma was first isolated from a human metastatic prostate adenocarcinoma found in a lymph node), 5637 (Primary Tumor of the Bladder), and ACHN (Metastatic Renal Adenocarcinoma) were obtained from Pishtaz Teb Zaman Diagnostics and the National Cell Bank of the Pasteur Institute of Iran. The PC3 cells were cultured in RPMI-1640 medium complemented with 10% fetal bovine serum (FBS), streptomycin (100 Ig/mL) (Gibco BRL; Thermo Fisher Scientific, Waltham, MA, USA), and penicillin–streptomycin (100 IU/mL). LNCaP cell lines were cultured in RPMI-1640 medium with 10% FBS and penicillin/streptomycin (100 U/ml) in 100 × 20 mm^2^ cell culture dishes in a humidified incubator with 5% CO_2_ at 37 °C. Four human cancer cell lines were cultured in RPMI-1640 Medium (Gibco, Carlsbad, CA), with 10% fetal bovine serum (Gibco, Carlsbad, CA), 1000 units/ml Penicillin, and 100 μg/ml streptomycin (Gibco BRL, Grand Island, NY) (Fig. 7).

The cells in the exponential growth phase with approximately 70%–80% confluences were cultured for experimental purposes in a humidified atmosphere using a 5% CO_2_ incubator at 37 °C. The cultured cells were screened for Mycoplasma species using the GenProb detection kit (Gen-Probe, San Diego, CA, USA) according to the manufacturer’s instructions.

### Cell viability assay and cell counting through tetrazolium test (MTT)

The MTT Cell Proliferation Assay is employed for assessing the rate of cellular proliferation and, conversely, when metabolic events lead to apoptosis or necrosis, the reduction in cell viability. The number of assay steps has been minimized as much as possible to expedite sample processing. To measure the metabolic activity of prostate cancer cell lines, we used the MTT Assay test^59^ (3-[4,5-dimethylthiazol-2-yl]-2,5 diphenyl Tetrazolium Bromide, Sigma-Aldrich, St. Louis, MO, USA). At first, both prostate cancer cell lines and their normal cell lines were seeded at three counts, 100, 250, and 500 per well in 96-well plates treated for 48 h at 37 °C with 5% CO_2_ saturation. For the MTT solution, 5 mg of MTT powder was dissolved in 10 ml RPMI-1640 without any serums to make a solution with a 0.5 mg/mL concentration. 100 μl of MTT solution was added to the cells and incubated for 4 h at 37 °C to metabolize this solution.

**Figure 8:**
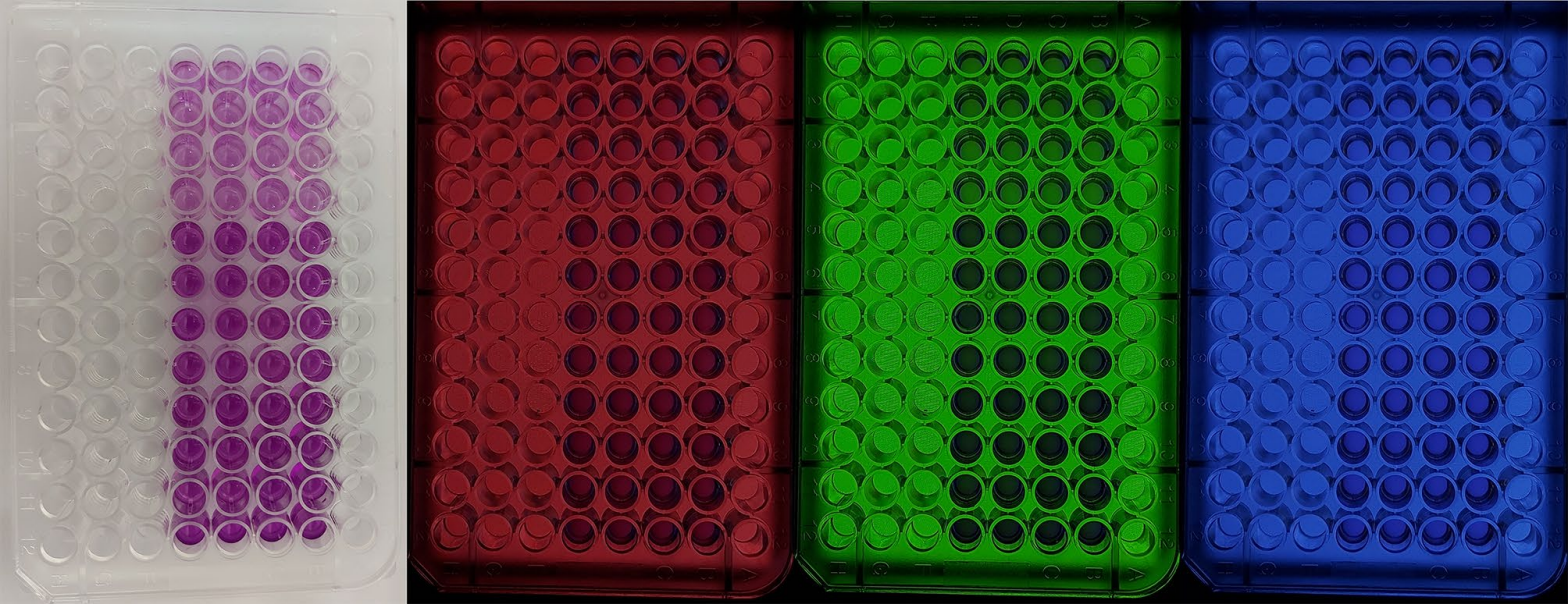
A microliter plate after an MTT assay. Increasing amounts of cells resulted in increased purple coloring.

Next, 100 µl of Dimethyl Sulfoxide was used to dissolve the formed Formazan crystals, which then formed a purple-colored solution. The optical density was afterwards read with an ELISA microplate reader ((MPR4 +), Hyperion, Medizintechnik GmbH & Co.KG, Germany)) at 570 nm wavelength (Fig. 8).

The %Viable cells % Death cells are calculated using as in trypan blue stain assay with following formulas:

%Viable cells = Total number of viable cells per milliliter of aliquot/Total number of cells per milliliter of aliquot × 100.

% Death cells = Total number of Dead cells per milliliter of aliquot/Total number of cells per milliliter of aliquot × 100.

%Viability = Mean OD sample / Mean OD control × 100.

## Conclusions and future prospective

We have developed a handheld, reasonably priced smartphone-based colorimetric microplate reader for immediate, simultaneous testing of multiple subjects, and portable determination of enzyme-linked immunosorbent assays. It uses a 3D-printed grip to illuminate a 96-well plate using an LCD array. Red, green, and blue color space images were processed to extract the appropriate colors, and the RGB values were utilized to assess the concentrations of human cancer cell lines. By integrating an Image Processing application and incorporating machine learning regression algorithms, the sensitivity of the portable ELISA was significantly enhanced and the optical density was successfully estimated. To evaluate its performance, the results were compared to those obtained from benchtop FDA-certified clinical plate readers: Epoch and Tecan.

We obtained an overall accuracy of 93.580%, 97.580%, and 97.288% for the HE4, PC3, and 5637 tests, respectively.

Although the specific focus of this research lies in cancer detection, there is potential for its application in a broader context. By incorporating samples from different viruses, bacteria, or any clinical or laboratory tests currently conducted using ELISA, the research can be expanded to encompass a wider range of applications. Furthermore, conducting actual clinical tests and implementing them on cancer-free samples would contribute to establishing its clinical validity.

This device can make a massive difference in rapid diagnostics, increase the level of healthcare, and provide extensive screening in disadvantaged and less developed areas.

### Supplementary Information

Supplementary Information.

## Data Availability

All data generated or analyzed during this study are included in this published article and its Supplementary Files.
