# Supplementary material for: A digital image colorimetry system based on smart devices for immediate and simultaneous determination of enzyme-linked immunosorbent assays
Source: Sci Rep. 2024 Jan 31;14:2587. doi: 10.1038/s41598-024-52931-6 (PMC10830485; doi:10.1038/s41598-024-52931-6)
Supplement: Supplementary file 1 — Supplementary Information. [file 41598_2024_52931_MOESM1_ESM.pdf]

A Digital Image Colorimetry System Based on Smart Devices for Immediate and Simultaneous Determination of Enzyme-Linked Immunosorbent Assays

Shaghayegh Mirhosseini, Aryanaz Faghih Nasiri, Fatemeh Khatami, Akram Mirzaei, Seyed Mohammad Kazem Aghamir, Mohammadreza Kolahdouz

Data

	R	G	B	sum		R	G	B	sum		R
G	68.46773163	174.566262	74.68792332	317.7219169	B	0.378913738	0.16485623	200.8446006	201.3883706	R	122.0199361
	69.98260584	184.0373045	69.50661853	323.5265288		0.385844	0.189804179	201.9386282	202.5142763		124.3651679
	65.33026941	166.4538827	70.36080296	302.1449551		0.317591125	0.151505547	204.3454834	204.81458		119.0180666
	65.81934217	169.5591556	68.29572901	303.6742268		0.364948454	0.203927344	200.9596465	201.5285223		118.2774669
	60.27729227	142.7880427	72.12026633	275.1856013		0.462616174	0.162435844	196.5765016	197.2015536		110.2014149
	53.79606684	126.5452995	62.68216437	243.0235307		0.40968512	0.198135728	189.6995567	190.3073775		102.5672388
	50.73366528	122.2960065	63.33665278	236.3663246		0.441012092	0.158317244	191.7230972	192.3224266		100.4175389
	52.80369783	127.8312731	62.66085578	243.2958267		0.475752773	0.164289488	191.7683043	192.4083465		101.5892235
	50.66026146	116.7364939	53.81571901	221.2124744		0.168215467	0.282406678	165.61679	166.0674122		94.276894
	37.34689376	73.72772944	51.09606666	162.1706899		0.252542581	0.270064943	165.1366254	165.6592329		80.17277294
	33.65169944	68.64385586	50.27623053	152.5717858		0.213254519	0.24417415	167.130158	167.5875867		77.72286007
	38.18848389	82.64405705	47.72815637	168.5606973		0.262968833	0.233703117	163.239197	163.735869		81.17062863
	31.20865749	60.74346699	39.23279473	131.1849192		0.353879912	0.314582087	129.004987	129.673449		68.25932575
	16.28124485	25.0304627	33.71612053	75.02782809		0.305121027	0.282726824	124.7788572	125.3667051		53.78379713
	15.35033231	23.86669223	29.9525818	69.16960634		0.403578275	0.332140575	122.9568051	123.692524		52.19527157
	20.80516355	37.80560114	29.63526966	88.24603435		0.343507275	0.249753856	121.6073734	122.2006345		57.98763811
	21.56949311	35.34407849	31.17893016	88.09250175		0.329829479	0.299929923	105.0474188	105.6771782		55.84839991
	18.50409234	28.20188877	26.64973767	73.35571878		0.289401889	0.259601259	107.3743966	107.9233998		53.30409234
	15.14876907	21.93399789	23.98640689	61.06917384		0.390877511	0.289382269	101.7420329	102.4222927		48.83159644
	12.85441665	20.24389186	17.47086887	50.56917739		0.287407836	0.337140379	91.88651149	92.51105971		44.86439208
	13.54552879	20.34177215	22.25683953	56.14414047		0.373621886	0.262964475	88.06043283	88.69701919		46.16986525
	8.210636079	10.93091762	16.34827946	35.48983316		0.304868524	0.300963291	80.40354074	81.00937256		37.18667014
	15.76660589	20.92966918	18.41755666	55.11383173		0.376204114	0.320229107	91.17365269	91.87008592		46.82452486
	13.29958014	21.81501605	15.24944431	50.3640405		0.407508027	0.314151642	85.87009138	86.59175105		44.94986416

G	B	sum
36.35271565	56.16638978	214.5390415
37.10961602	53.24734712	214.7221311
31.98626519	52.16798732	203.1723191
32.15670103	50.09297987	200.5271478
27.12609238	52.45581911	189.7833264
22.26304422	46.0954871	170.9257701
20.5440504	45.308302	166.2698913
21.22694136	44.52646593	167.3426307
20.10001575	40.49566861	154.8725784
10.28489156	36.91140792	127.3690724
9.035239286	35.46765943	122.2257588
11.86687797	34.31664025	127.3541469
8.146020347	28.3433074	104.7486535
2.981228388	24.50008233	81.26510785
2.62172524	22.6371885	77.4541853
4.624767531	22.12876053	84.74116617
4.54519972	22.12099977	82.51459939
3.413641133	20.44281217	77.16054565
3.053315209	17.96949101	69.85440266
2.943761746	14.44455689	62.25271071
2.849326256	16.74642711	65.76561862
1.744337412	13.26451445	52.195522
3.207758396	14.79744858	64.82973184
3.127932823	12.91207706	60.98987404

Epoch	Tecan	Device's OD
0.171	0.167862868	0.173915717
0.16	0.168727755	0.158200954
0.175	0.18431758	0.175354077
0.176	0.19430836	0.175805944
0.226	0.213496837	0.222885754
0.273	0.261155085	0.276796047
0.275	0.27039092	0.271841207
0.255	0.270746744	0.255613092
0.406	0.342802018	0.403885372
0.442	0.427445774	0.447012568
0.445	0.442602365	0.437646276
0.429	0.437648254	0.441853826
0.68	0.632307555	0.659812135
0.78	0.768203421	0.757740702
0.81	0.802349697	0.773883914
0.704	0.768444923	0.747860891
0.888	0.85545274	0.864622101
0.893	0.876763212	0.877277795
0.874	0.961285104	0.951810756
1.012	1.084840287	1.06385428
1.087	1.069768067	1.094200639
1.274	1.259913914	1.22342905
1.082	1.063777641	1.059005315
1.127	1.133645597	1.118713222

RGB Plate Calibration Data

	R	G	B	sum		R	G	B	sum		R	G	B
G	1.173601	161.9699	105.1736	268.3171	B	1.126255	83.45337	240.7834	325.363	R	176.7475	28.27547	80.74032
	1.208071	159.4968	87.32787	248.0328		0.948298	64.84868	200.4943	266.2913		173.034	26.95334	35.16772
	0.963556	158.9467	75.72533	235.6356		0.787556	57.92356	143.5342	202.2453		173.2436	25.72356	7.298667
	1.075237	160.748	68.93207	230.7553		0.720964	55.73484	108.5931	165.0489		172.0256	25.63915	3.925493
	1.204366	160.2141	65.83323	227.2517		0.61795	54.11886	91.68163	146.4184		166.4554	32.49363	4.36507
	1.001213	162.5052	65.53305	229.0394		0.55185	54.57126	79.78229	134.9054		164.7029	33.11886	4.069739
	1.368921	161.0822	62.26775	224.7188		0.631638	53	79.53997	133.1716		166.4142	32.81666	5.953605
	1.233291	162.1564	103.8045	267.1942		1.124842	85.06431	242.6456	328.8348		178.1488	29.05044	83.72509
	1.301462	160.5624	87.55568	249.4196		0.912261	66.28796	199.7559	266.9561		175.6749	26.59505	32.64792
	1.186267	159.9759	76.22644	237.3886		0.753835	57.70489	145.851	204.3097		174.2148	25.34624	8.630387
	0.942498	160.1183	71.10112	232.1619		0.740251	54.3503	113.0991	168.1897		172.0423	26.48249	3.035691
	1.387508	161.5106	65.97029	228.8684		0.636143	53.68648	94.17041	148.493		174.4318	26.67314	3.544572
	1.325605	161.9964	61.03452	224.3565		0.583205	52.37094	85.24781	138.202		170.7156	29.97836	2.788253
	1.951571	160.1128	63.23184	225.2962		0.612571	51.31994	84.89284	136.8253		167.7161	32.5662	4.193715
	1.134983	161.4826	108.3375	270.955		1.035996	85.05962	241.7064	327.802		175.2508	32.22835	82.03262
	1.345778	160.1511	89.55111	251.048		1.076444	67.49422	200.136	268.7067		174.3342	29.89867	35.49867
	1.002191	162.4098	78.36158	241.7736		0.831264	59.2542	146.7093	206.7947		170.9028	31.42148	8.309715
	1.355367	162.262	69.43663	233.054		0.668284	56.7829	112.7526	170.2038		169.8866	33.06792	2.65373
	1.680577	162.5698	75.08913	239.3395		0.622875	53.86502	98.23442	152.7223		174.1097	28.63936	3.317877
	1.810503	162.4333	64.93348	229.1772		0.608753	53.00306	87.90328	141.5151		168.9466	32.7046	3.344858
	1.366306	161.4982	65.42143	228.2859		0.678516	51.22154	84.87378	136.7738		171.0263	32.05873	1.985059
	0.740316	160.5136	107.4735	268.7274		0.902439	87.38881	242.4849	330.7762		176.3572	33.1076	85.09756
	0.765174	160.8488	92.01493	253.6289		0.827861	70.42488	200.6806	271.9333		174.3542	31.78109	36.75025
	0.875822	161.4456	79.02045	241.3419		0.804967	59.56757	152.7502	213.1227		174.5763	29.1702	7.11176
	1.746786	161.2532	73.8597	236.8597		0.827278	57.54891	112.9743	171.3505		171.3667	32.78256	3.61934
	1.682314	162.2987	70.7762	234.7572		0.790422	55.10953	103.0341	158.9341		170.7037	34.60313	6.532006
	2.442888	163.8941	72.67965	239.0166		0.811379	53.14311	88.39168	142.3462		170.5895	35.0442	4.393435
	2.677024	162.8354	67.9558	233.4683		0.778556	50.78556	87.53611	139.1002		177.4687	27.83589	2.996061
	0.677188	158.8422	110.7016	270.2209		0.855093	88.77905	243.7116	333.3458		175.8178	35.05165	91.48063
	0.974222	160.7902	92.304	254.0684		0.989333	71.51289	200.8044	273.3067		174.7209	33.60267	40.67111
	0.79401	161.3389	78.2637	240.3966		0.712929	62.25639	150.2915	213.2608		175.3338	31.49671	7.080351
	1.192845	162.4751	75.26831	238.9363		0.722191	58.62549	120.7658	180.1135		176.1755	33.57071	3.154835

1.883589	163.7063	78.42363	244.0136
3.40744	162.9103	63.07702	229.3947
2.489331	162.2371	66.55334	231.2798
0.626973	161.2783	113.6011	275.5065
1.324378	162.2647	93.25174	256.8408
0.731921	161.8656	78.25566	240.8532
1.183343	162.5472	70.91671	234.6473
2.094832	162.9175	69.06401	234.0763
2.311556	163.5259	65.89588	231.7334
2.075711	159.3641	71.27834	232.7182
0.778325	160.2463	115.7701	276.7947
1.255721	163.0667	93.79502	258.1174
0.853908	162.8459	78.65961	242.3594
1.569033	162.7865	72.85355	237.2091
2.387387	162.9782	69.93646	235.302
2.871335	158.2534	65.64508	226.7698
2.493599	159.7008	61.39782	223.5922
1.148802	161.7175	118.8739	281.7402
1.459701	164.3035	96.76418	262.5274
1.026297	162.6932	80.66326	244.3828
1.667677	163.0679	70.87083	235.6064
1.872231	163.2586	67.20453	232.3354
1.133952	161.3235	66.87326	229.3308
1.919393	158.559	59.28402	219.7624

0.754923	55.77856	104.0346	160.5681
0.808753	54.7663	91.27527	146.8503
0.843054	52.4808	89.60503	142.9289
0.879484	92.71019	245.5294	339.1191
0.976119	74.53134	203.5781	279.0856
0.718773	66.31556	154.29	221.3243
0.815539	62.95808	119.4097	183.1833
0.895211	59.28023	103.6482	163.8236
0.917926	56.58065	96.40465	153.9032
0.817505	53.7965	90.59037	145.2044
0.99179	94.28079	245.1511	340.4236
0.814925	79.28756	206.6129	286.7154
0.81008	68.53908	157.5698	226.9189
0.946339	64.65456	125.4969	191.0978
0.991465	63.11143	103.3476	167.4505
1.064333	56.786	97.70635	155.5567
0.991939	55.79422	92.33618	149.1223
1.093317	98.1034	246.7074	345.9042
0.98806	82.23383	208.3841	291.606
0.855369	71.41928	158.2082	230.4828
1.027896	68.15464	118.5797	187.7623
0.931994	64.69243	102.694	168.3184
0.702731	60.65688	92.84905	154.2087
0.865813	56.43054	85.30441	142.6008

174.6114	34.65777	3.727352
173.733	34.72779	3.256018
172.5102	37.21622	3.25652
179.34	38.77188	102.2037
176.6328	35.34627	43.14726
179.7049	33.52593	9.102264
178.242	36.29514	2.847401
174.2821	38.75154	3.614509
176.6684	33.3038	5.048591
168.9799	38.26258	3.787746
179.8604	41.65846	108.757
181.0189	40.36119	48.74726
180.4945	37.89627	14.52228
180.0945	38.42538	5.580771
181.4021	36.83973	3.753912
169.9002	38.52123	6.367615
174.6245	37.74016	3.479374
181.0391	44.92938	114.8638
183.3114	43.18109	53.92239
180.8517	40.90212	16.20161
181.6938	41.15343	6.359612
178.185	42.06801	4.672334
176.6662	36.79134	3.405461
174.7895	38.33618	3.210526

sum	Pos	OD1	OD2	OD3	OD(ave)	OD-PRED(BB)
285.76327	A1	0.042	0.042	0.042	0.042	0.09789813
235.15511	A2	0.536	0.538	0.542	0.538666667	0.247136544
206.26578	A3	1.123	1.13	1.134	1.129	0.915185474
201.59021	A4	1.661	1.67	1.677	1.669333333	2.04309639
203.31413	A5	2.074	2.084	2.093	2.083666667	3.013689222
201.89145	A6	2.534	2.546	2.557	2.545666667	3.961656841
205.18446	A7	3.152	3.168	3.18	3.166666667	3.983783564
290.92434	B1	0.042	0.042	0.042	0.042	0.093796172
234.91789	B2	0.549	0.554	0.557	0.553333333	0.251366749
208.19138	B3	1.129	1.135	1.139	1.134333333	0.867727433
201.56048	B4	1.669	1.677	1.685	1.677	1.842087172
204.64948	B5	2.079	2.091	2.1	2.09	2.846135744
203.48223	B6	2.556	2.569	2.579	2.568	3.493979865
204.47604	B7	3.124	3.138	3.16	3.140666667	3.522603016
289.51181	C1	0.042	0.042	0.041	0.041666667	0.095843028
239.73156	C2	0.545	0.55	0.554	0.549666667	0.249180326
210.63404	C3	1.143	1.148	1.151	1.147333333	0.850777359
205.60825	C4	1.672	1.677	1.684	1.677666667	1.85681892
206.06698	C5	2.065	2.077	2.09	2.077333333	2.592321901
204.99606	C6	2.593	2.606	2.613	2.604	3.287105519
205.07007	C7	3.145	3.158	3.181	3.161333333	3.524146725
294.56241	D1	0.042	0.042	0.042	0.042	0.094143286
242.88557	D2	0.547	0.551	0.555	0.551	0.246080736
210.85829	D3	1.119	1.125	1.128	1.124	0.740484521
207.76859	D4	1.671	1.679	1.686	1.678666667	1.847380936
211.83879	D5	2.083	2.092	2.1	2.091666667	2.32155184
210.02713	D6	2.546	2.558	2.563	2.555666667	3.250412215
208.30066	D7	3.162	3.176	3.183	3.173666667	3.314963764
302.35007	E1	0.042	0.042	0.042	0.042	0.091526036
248.99467	E2	0.547	0.55	0.553	0.55	0.245381253
213.91088	E3	1.136	1.142	1.145	1.141	0.783535832
212.90106	E4	1.664	1.671	1.679	1.671333333	1.544479544

212.9965	E5	2.087	2.096	2.104	2.095666667	2.268778604
211.71685	E6	2.553	2.565	2.571	2.563	3.041968006
212.98293	E7	3.142	3.164	3.176	3.160666667	3.161017836
320.31564	F1	0.042	0.042	0.042	0.042	0.08778081
255.12637	F2	0.547	0.55	0.552	0.549666667	0.230226245
222.33309	F3	1.122	1.127	1.132	1.127	0.714736066
217.38457	F4	1.675	1.683	1.689	1.682333333	1.593376157
216.64817	F5	2.082	2.093	2.104	2.093	2.289017571
215.02082	F6	2.57	2.576	2.586	2.577333333	2.703668745
211.0302	F7	3.176	3.188	3.204	3.189333333	3.090233426
330.27586	G1	0.043	0.043	0.043	0.043	0.088547477
270.12736	G2	0.548	0.551	0.554	0.551	0.214714508
232.91308	G3	1.13	1.136	1.141	1.135666667	0.662837816
224.10061	G4	1.648	1.66	1.669	1.659	1.385339677
221.99573	G5	2.121	2.131	2.139	2.130333333	2.304888178
214.78906	G6	2.548	2.56	2.567	2.558333333	2.623977372
215.844	G7	3.131	3.151	3.164	3.148666667	2.968689372
340.83228	H1	0.042	0.042	0.041	0.041666667	0.085435935
280.41493	H2	0.544	0.547	0.55	0.547	0.206149336
237.95544	H3	1.127	1.131	1.138	1.132	0.653182571
229.20679	H4	1.648	1.662	1.675	1.661666667	1.624063965
224.9253	H5	2.084	2.095	2.104	2.094333333	2.339774015
216.86296	H6	2.544	2.554	2.562	2.553333333	2.933900193
216.33618	H7	3.157	3.168	3.181	3.168666667	3.489437529

OD-PRED(sum)	OD-PRED(GB_BB_RB)	OD-PRED(GB_BG_BB_Bsum_RB)
0.099855492	0.081473593	0.082940943
0.474312262	0.427044557	0.413445673
1.542744785	1.483617425	1.447226747
2.518749728	2.172091145	2.12207509
3.127981469	2.463428831	2.352071587
3.408464673	2.706194871	2.575758008
3.623075237	2.623971201	2.510563106
0.092878464	0.074632577	0.075860431
0.459995906	0.459732229	0.44620501
1.428220795	1.402357913	1.349966128
2.381715872	2.128646581	2.077356069
2.924498562	2.474229154	2.366399378
3.560960079	2.772306904	2.605085795
3.495826818	2.637743872	2.489333894
0.088519694	0.07661625	0.076947344
0.413191246	0.41855791	0.425430941
1.24214356	1.388339327	1.377429339
2.186234029	2.178371192	2.107160127
2.246612698	2.289875451	2.208492931
3.082419972	2.616821324	2.489323317
3.271727875	2.768009208	2.674240709
0.08465618	0.070350057	0.068541708
0.36677207	0.396775974	0.384126575
1.180032694	1.369899003	1.34787201
1.953554524	2.06168256	2.096016348
2.167357103	2.078129758	2.069796089
2.365166962	2.417329813	2.448985762
2.777721945	2.601239997	2.57288366
0.072969363	0.057295647	0.055174865
0.333135801	0.354942931	0.357920793
1.156706829	1.401480659	1.365479555
1.626157932	1.9599499	1.940172636

1.751672452	2.129895655	2.141918434
2.693011291	2.590450616	2.59946414
2.645974329	2.566707541	2.589449039
0.050006443	0.041278836	0.040179254
0.27753631	0.323097016	0.327642255
0.961019785	1.288209224	1.271971138
1.630139171	2.04886393	2.108950205
1.981423677	2.266923233	2.367874792
2.316190123	2.337017988	2.424207251
2.580293025	2.441006211	2.475836913
0.042623229	0.034058839	0.034153612
0.210062051	0.269849892	0.265545597
0.778658049	1.080336726	1.091288042
1.328161491	1.798202825	1.915586511
1.75245253	2.251082386	2.457285455
2.525638049	2.235807071	2.401356773
2.814448474	2.581270665	2.716932978
0.032295958	0.027908167	0.029064251
0.162262888	0.226749375	0.235308645
0.680778427	1.014175016	1.050422354
1.327132931	1.870046886	2.072825989
1.779173512	2.241239118	2.414818142
2.368565041	2.493443447	2.51359269
3.201766269	2.768010037	2.840301251

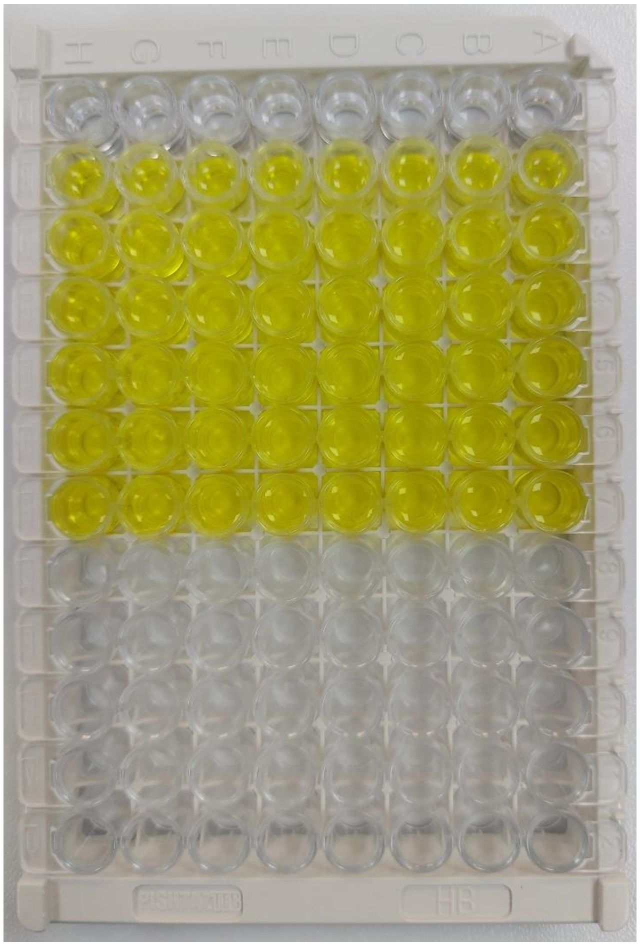
H G F E D C B A
HB

Cross Validation Data

	Missing Input	Output
**0**	0.257548475	0.293745856
**1**	0.276928299	0.313810669
**2**	0.290377927	0.291445411
**3**	0.29254172	0.30396156
**4**	0.313125594	0.26502235
**5**	0.311146602	0.263130769
**6**	0.332082147	0.351691022
**7**	0.335707518	0.351391413
**8**	0.322785172	0.322228867
**9**	0.262568792	0.293995586
**10**	0.262568792	0.293995586
**11**	0.305108861	0.283425264
**12**	0.283756923	0.271087962
**13**	0.303061348	0.291357007
**14**	0.31896704	0.295784768
**15**	0.320883503	0.311890147
**16**	0.328404475	0.344988806
**17**	0.405315304	0.396447568
**18**	0.249769661	0.283000842
**19**	0.974384687	0.921007094
**20**	0.267467086	0.310366578
**21**	0.381908777	0.352808248
**22**	1.498540475	1.563265344
**23**	0.348012344	0.310858377
**24**	1.556318281	1.602853979
**25**	0.423110919	0.398129758
**26**	0.401061631	0.39279291
**27**	0.249769661	0.283000842
**28**	1.627882054	1.662606485
**29**	0.262568792	0.293995586
**30**	0.349724355	0.306078756
**31**	1.74673397	1.723945618
**32**	0.288192925	0.28055906
**33**	1.796830685	1.730058277
**34**	0.267467086	0.310366578
**35**	0.689622405	0.700608946
**36**	0.241661715	0.255566717
**37**	1.451956566	1.481995214
**38**	0.433593793	0.402114543
**39**	0.28150467	0.279466586
**40**	1.784222672	1.729594649
**41**	0.311146602	0.263130769
**42**	1.667538381	1.690031735
**43**	0.276928299	0.313810669
**44**	0.697318154	0.714124277
**45**	0.241661715	0.255566717

**46**	1.406964469	1.42692177
**47**	0.511920353	0.426667357
**48**	0.269872968	0.317670925
**49**	1.639568174	1.67129208
**50**	0.322785172	0.322228867
**51**	1.6411637	1.672441864
**52**	0.274602736	0.318316294
**53**	0.553352508	0.529290034
**54**	0.236053336	0.228582856
**55**	0.260074413	0.288956346
**56**	0.249769661	0.283000842
**57**	0.283756923	0.271087962
**58**	0.315088427	0.270857365
**59**	0.311146602	0.263130769
**60**	0.307138632	0.273394739
**61**	0.290377927	0.291445411
**62**	0.298911328	0.313531014
**63**	0.283756923	0.271087962
**64**	1.724497607	1.717463908
**65**	0.244403338	0.266304558
**66**	0.315088427	0.270857365
**67**	1.772940198	1.728573572
**68**	0.303061348	0.291357007
**69**	1.793182186	1.729997167
**70**	0.29254172	0.30396156
**71**	1.104054861	1.07876789
